# Retraction Note: Identify and measure the degree of over-prevention behaviors in the post-COVID-19 era in China

**DOI:** 10.1186/s12889-021-12352-w

**Published:** 2021-12-10

**Authors:** Rongyang Ma, Hong Wu, Zhaohua Deng

**Affiliations:** 1 School of Medicine and Health Management, Huazhong University of Science and Technology, No. 13 Hangkong Road, Qiaokou District, Wuhan, 430030 China; 2 School of Management, Huazhong University of Science and Technology, Wuhan, China

**Retraction Note to: BMC Public Health 21, 1743 (2021)**

**https://doi.org/10.1186/s12889-021-11823-4**

The Editors have retracted this article because this study had not received any ethics approval.

None of the authors have responded to any correspondence from the editor/publisher about this retraction.

